# Simulation-Based Training for Colonoscopy: Establishing Criteria for Competency

**DOI:** 10.1097/MD.0000000000000440

**Published:** 2015-01-30

**Authors:** Louise Preisler, Morten Bo Søndergaard Svendsen, Nikolaj Nerup, Lars Bo Svendsen, Lars Konge

**Affiliations:** From the Department of Surgical Gastroenterology and transplantation, Rigshospitalet, and Copenhagen University (LP,LBS), Centre for Clinical Education (CEKU) and Copenhagen University (MBSS,LK), Department of Surgical Gastroenterology, University hospital of Herlev (NN), Copenhagen University Hospital, Herlev, The Capital Region of Denmark.

## Abstract

The aim of this study was to create simulation-based tests with credible pass/fail standards for 2 different fidelities of colonoscopy models.

Only competent practitioners should perform colonoscopy. Reliable and valid simulation-based tests could be used to establish basic competency in colonoscopy before practicing on patients.

Twenty-five physicians (10 consultants with endoscopic experience and 15 fellows with very little endoscopic experience) were tested on 2 different simulator models: a virtual-reality simulator and a physical model. Tests were repeated twice on each simulator model. Metrics with discriminatory ability were identified for both modalities and reliability was determined. The contrasting-groups method was used to create pass/fail standards and the consequences of these were explored.

The consultants significantly performed faster and scored higher than the fellows on both the models (*P* < 0.001). Reliability analysis showed Cronbach α = 0.80 and 0.87 for the virtual-reality and the physical model, respectively. The established pass/fail standards failed one of the consultants (virtual-reality simulator) and allowed one fellow to pass (physical model).

The 2 tested simulations-based modalities provided reliable and valid assessments of competence in colonoscopy and credible pass/fail standards were established for both the tests. We propose to use these standards in simulation-based training programs before proceeding to supervised training on patients.

## Abstract 2

Supplemental Digital Content is available in the text

## INTRODUCTION

The need for skilled endoscopists is growing as national screening programs for colorectal cancer is becoming more common. Existing centers will have to perform more procedures while at the same time instruct and supervise more trainees. Simulation-based training programs have the potential to alleviate this problem by speeding up the early training.^1–3^ A Cochrane review from 2012 found that virtual-reality endoscopy training is useful as an effective supplement to early conventional endoscopy training (apprenticeship model).^4^ However, there is no agreed standard as how to establish and run these programs, which equipment to use, which length of training to apply, and whether or not there should be instructors present during training. Not all trainees learn at the same pace.^5,6^ Slow learners fail to meet the intended goals and quick learners risk wasting valuable time in fixed-length training programs. Training to a criterion would allow for unsupervised practice. Furthermore, it would ensure that all trainees reach a defined level of competency before practicing on patients. Such a criterion would have to be defined specifically for each simulator and must be based on reliable and valid assessments, using a credible standard setting method.^7^ Traditionally, competence in colonoscopy has been defined by performance of a certain number of procedures. The exact number varies from 100 to 200,^2,8^ and research suggests that 140, 275, or even 500 procedures are needed to ensure competency.^9–11^ Those differences underpin the impossible notion of establishing competency based on numbers. Actually, Leyden et al^12^ found that the number of procedures previously performed did not predict quality outcomes. In an era of competency-based education, certification must be based on robust assessment methods and credible standards. Two such tools have recently been introduced. The Mayo Colonoscopy Skills Assessment Tool (MCSAT) consists of 14 items of which only 5 assess motor skills, whereas the Direct Observation of Procedural Skills (DOPS) has 20 items in 4 main domains. Only 1 domain deals with endoscopic skills during insertion and withdrawal.^13^ MCSAT has been described as a unique instrument to assess colonoscopy skills in trainees,^11^ whereas DOPS was developed to assess colonoscopy skills of senior endoscopists. Both tools have been tested for reliability and validity, and credible standards have been established using recognized methods. A further development of DOPS including polypectomy is also validated.^14^ However, these results from clinical settings cannot be generalized to a simulation-based environment and so far no attempts have been made to gather valid evidence on the use of either tool on simulated procedures.

Several studies use the metrics provided by the virtual-reality simulators to assess competence. These metrics arise from completely standardised tasks (as opposed to the very heterogeneous patient encounters) and are unbiased (as opposed to assessment done by direct observation by faculty that are prone to all sorts of bias such as the three “isms”—sexism, racism, and ageism).^15^ However, reliability and validity of these metrics must be carefully explored before use, as not all possess discriminatory ability. Furthermore, all validity is specific—there is no such thing as “a valid simulator.” Specific test programs have to be designed specifically for each simulator, and the psychometric properties of these tests must be explored. This is also true for other simulation modalities such as bovine or rubber phantoms.

Traditionally, simulation-based assessment has been used for formative assessment. More than 100 studies have described methods for objective skills assessment in surgery and valid feedback when it comes to measuring progress of training, but few have been developed with the purpose of standard setting.^16^ A standard determines whether a given score is good enough and is necessary for summative assessment. Pass/fail decisions should never be based on a single performance because of the possibility of rater errors and variability of performance. A meaningful certification should be based on normative criteria and mirror expectations of the clinical setting.^17^

The aims of this study were to create specific tests based on a virtual-reality simulator and a phantom model, respectively, and explore the reliability and validity of these tests and furthermore to set credible pass/fail standards and explore the consequences of these.

## METHODS

The study was designed as a prospective nonrandomized study. We created 2 simulation-based tests for assessing competence in colonoscopy: 1 based on a virtual-reality simulator and 1 using a phantom model. The virtual-reality simulator provided preset metrics, whereas a specific assessment tool had to be created for the phantom simulator model. The tests as well as the assessment tool were created by the research group consisting of 2 content experts (experts in colonoscopy) and 1 expert in medical assessment. Both tests were administered to a group of novices and a group of experienced endoscopists, and the resulting metrics were tested for discriminatory ability to create a single aggregated score for each model. Finally, the contrasting groups’ method was used to establish credible passing scores for both the tests.

### Virtual-Reality Simulator Test

In this test, we used the GI Mentor virtual-reality simulator (Simbionix Corporation, Cleveland, OH). This model provides cases ranging from simple diagnostic procedures (case 1) to more challenging procedures including pathology (case 10). For this test, case 2 (easy) and case 9 (difficult) were chosen. The following metrics were analyzed: percentage of colonic mucosa visualized, time with clear view, time to reach cecum, intubation of terminal ileum, loop formation, “patient” discomfort, and hazardous tension to the bowel.

### Phantom Model Test

A simple and operational assessment tool was created specifically for the phantom model. Only core technical skills of colonoscopy were rated on a scale from 0 to 2 points (handling of the scope, safe scope advancement, extensive force used, visualized mucosa during withdrawal, and successful looping in rectum). For details, see Appendix 1, http://links.lww.com/MD/A166.

We used the Kagaku Colonoscope Training Model (Kyoto Kagaku Co Ltd, Kyoto, Japan) in combination with a standard colonoscope (Olympus CF180AL; Olympus Medical Systems Corp, Tokyo, Japan) with air insufflation, suction, and water, OEV203 monitor, and a ScopeGuide (Olympus Medical Systems Corp). The phantom model consisted of a flexible rubber colon tube inside a life-size mannequin. The colon tube in the phantom could be adjusted into 6 different positions using Velcro-strips and rubber bands to simulate a range from simple to more difficult procedures. A technical easy case and a more difficult case presenting loop formation in colon sigmoid were chosen for this study.

### Participants

Fifteen novices and 10 experienced consultants (gastroenterologists n = 2, colorectal surgeons n = 8) participated in the study. Novices were recruited from fellows in gastroenterology and gastrosurgical fellows during their first or second year of fellowship. The participants had to actively sign up for the test and no randomizing procedure was made. Fellows who previously had received formal simulator training or performed >2 colonoscopies in a clinical setting were excluded from the study. The experienced group had all >5 years of experience in colonoscopy and had performed more than 350 colonoscopies. All participants were recruited and tested between November 2012 and March 2013.

All participants signed a letter of informed consent and filled out a brief questionnaire, including demographics, such as gender, age, years of endoscopy experience, previous colonoscopy experience, and the number of colonoscopies performed the past year before entering the study.

### Ethics

There was no physical or psychological discomfort to the participants. The collected data was anonymized. Participants signed a letter of informed consent before entering the study.

## GATHERING DATA

### Virtual-Reality Simulator Test

Testing was conducted in a research laboratory in a medical simulation center. The arrangement of all equipment simulated that of an endoscopy suite. The novices were introduced to the functions of the colonoscope including holding the colonoscope, using the controls (dials, insufflation, suction, and water), manipulating the colonoscope tip, and torque steering. They had 30 minutes of supervised training before the test. The experienced group had 15 minutes to try out the simulator. All participants attempted the 2 cases in a standardized order and there was a maximum of 10 minutes allowed for each case.

### Phantom Model Simulator Test

The test setting was a fixed setup in a dedicated room and was not changed during the study period. Both groups were introduced to the phantom model and had 15 minutes to try it out. This test was conducted immediately following the test on the virtual-reality model without further introduction to either of the groups. Participants were instructed to treat the model as if it was a real patient and there was a maximum of 15 minutes allowed for each case. Assessment was done during the procedure using the created assessment tool.

### Statistical Analysis

We used a stepwise approach presented by Konge et al^18^ to collect validity evidence in the broadest sense (including reliability testing and standard setting).

Each simulator metric was tested and metrics showing discriminatory ability were combined into a single aggregated score for each model. Metrics with discriminatory ability were identified using a 2-way mixed analysis of variance (ANOVA) with cases as repeated measures variable and experience as grouping factor (either novices or experienced endoscopists). Ability to intubate terminal ileum (categorical variable) was analyzed using Fisher exact test. These metrics were used to create an aggregated score for each model. The aggregated score was created as average of the metrics with discriminating ability divided by the time spent. The models were separately explored in order to be able to compare them. Interrater reliability was analyzed by intraclass correlation coefficient (ICC), average measure (=Cronbach α; reliability of both cases combined), and single measure (reliability when assessing only 1 case). The performances of the 2 groups were compared for each of the 2 models by performing independent samples *t* test and Levene test for equality of variance. In order to improve the reliability, the participants performed 2 tasks on each model.

Statistical analysis was performed using a statistical software package (PASW, version 18.0; SPSS Inc, Chicago, IL). Differences were considered statistically significant for *P* values <0.05.

For standard setting, we chose the contrasting-groups method, 2 contrasting groups based on an external criterion: 1 group of noncompetent performers (fellows = novices) and 1 group of competent performers (consultants = experienced endoscopists).^15^ The standard was established for each test by graphing the 2 aggregated score distributions and finding the score that best discriminated between the 2 groups. The passing score was set at the intersection of the distributions and the consequences of the standard were explored for each test. According to the Danish rules, an ethical committee approval was not necessary for the study.

## RESULTS

Table 1 shows the demographics and experience of the participants.

**TABLE 1:**
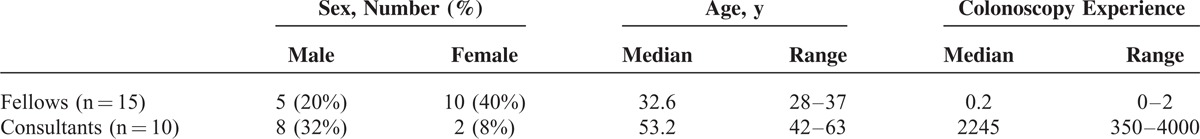
Demographics of the Participating Physicians

###  Virtual-Reality Assessment Tool

The following metrics had discriminatory ability: percentage of colonic mucosa visualized, percentage of time with clear view, intubation of terminal ileum, and time to reach cecum (Table 2). These metrics were combined to an aggregated score. Successful intubation of terminal ileum equaled 100%, and the mean percentage of the 3 scores was divided by the time to cecum. Other metrics such as loop formation, “patient” discomfort, and hazardous tension to the bowel did not show discriminatory ability and these were not included in the aggregated score.

**TABLE 2:**
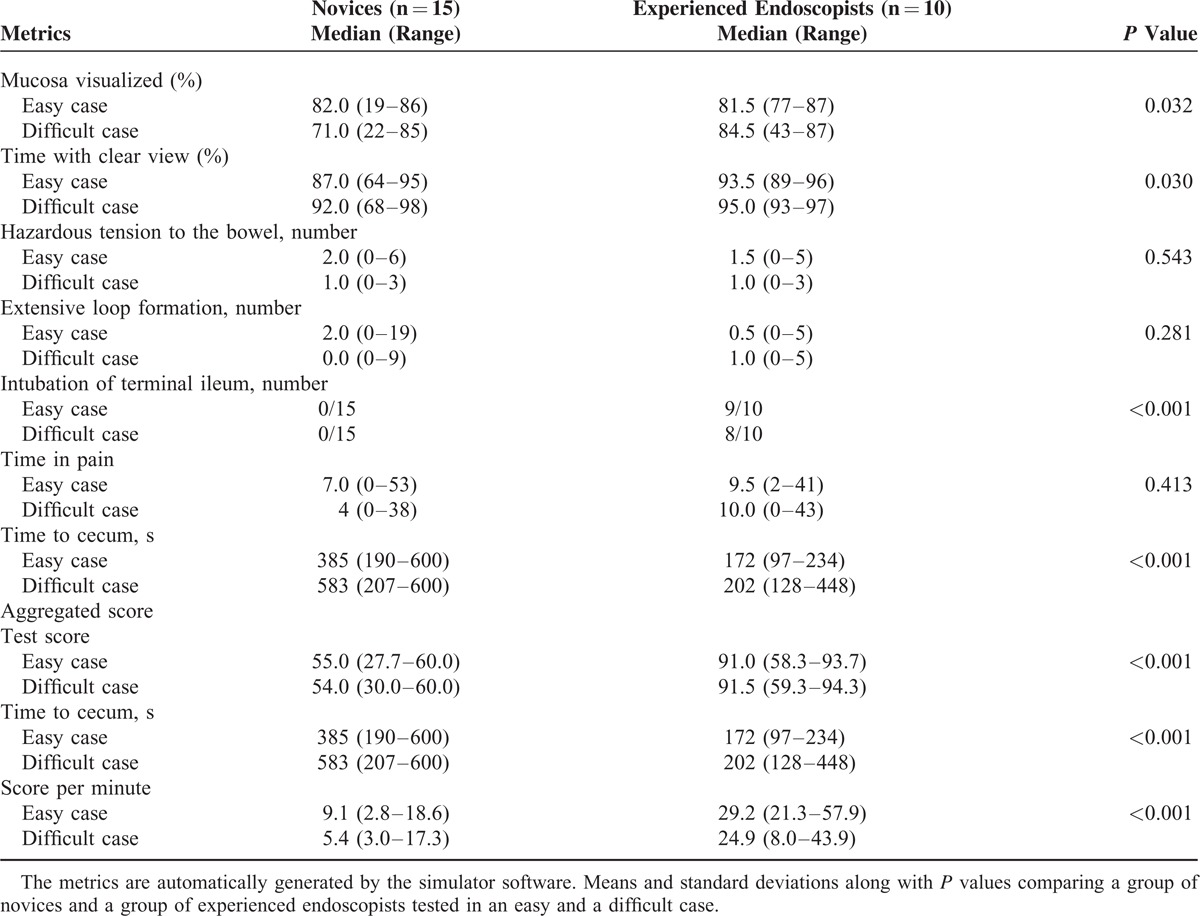
Metrics in the Virtual–Reality Simulator Test

Reliability of the aggregated score was tested showing an ICC, average measures = 0.80. Single measure showed a reliability of 0.67.

**FIGURE 1:**
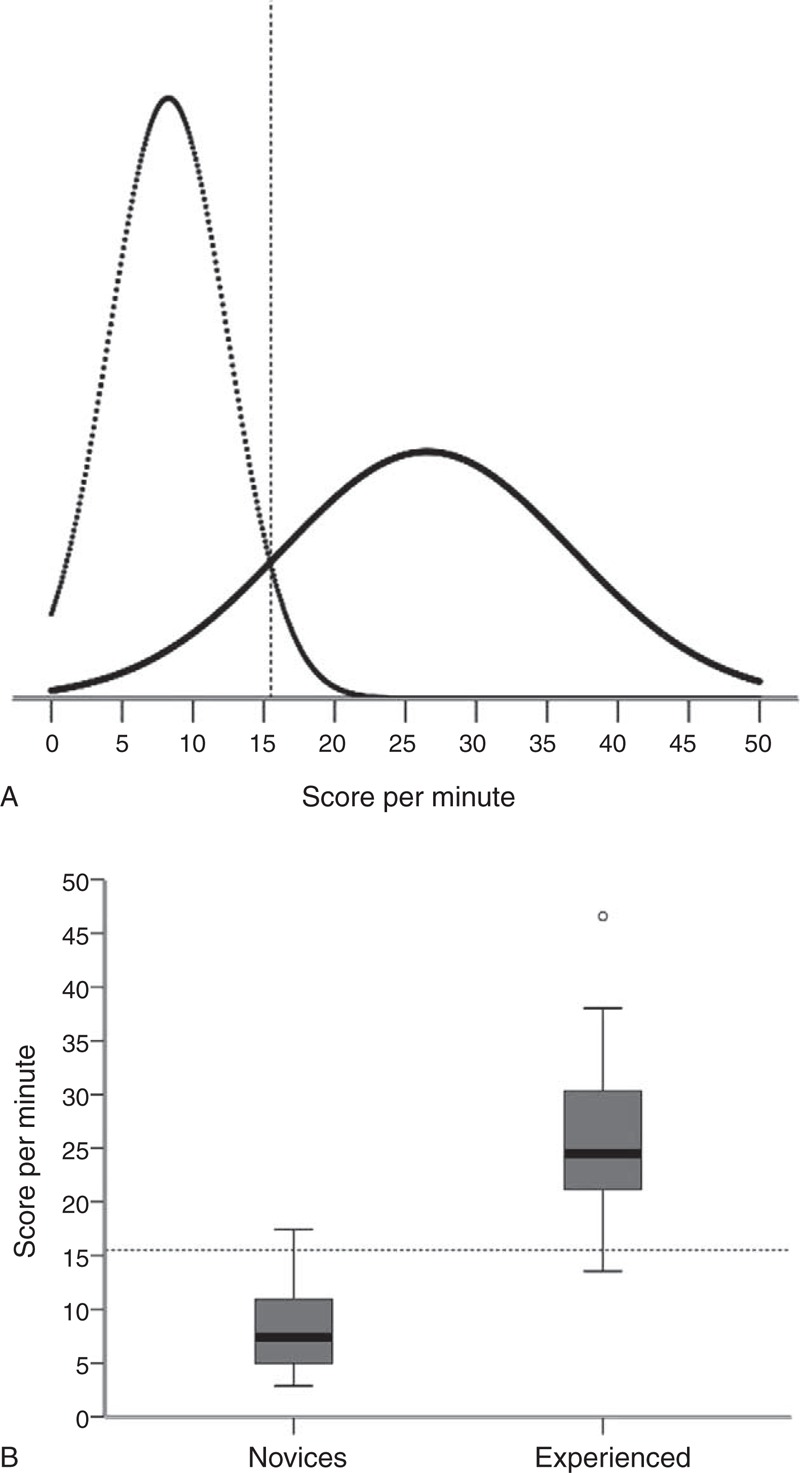
Virtual-reality simulator test. (A) Establishing a pass/fail standard using the contrasting-groups method. The distributions of scores of novices (dotted line, n = 15) and experienced endoscopists (solid line, n = 10) are shown. The pass score (15.5 points/min) was set at the intersection of the score distributions of the 2 groups. (B) Box plot showing the consequences of the established pass/fail criterion. One of the experienced endoscopists failed the test and one novice passed the test.

The mean aggregated score per minute in the novice group was 7.2 (standard deviation [SD] 1.1) and for the experienced group was 27.0 (SD 3.2), *P* < 0.001. The pass/fail score was set at the intersection of distributions = 15.5 points/minutes (Figure 1A), and the consequences of this standard were explored showing that 1 of the consultants did not pass the test (Figure 1B).

### Phantom Assessment Tool

**FIGURE 2:**
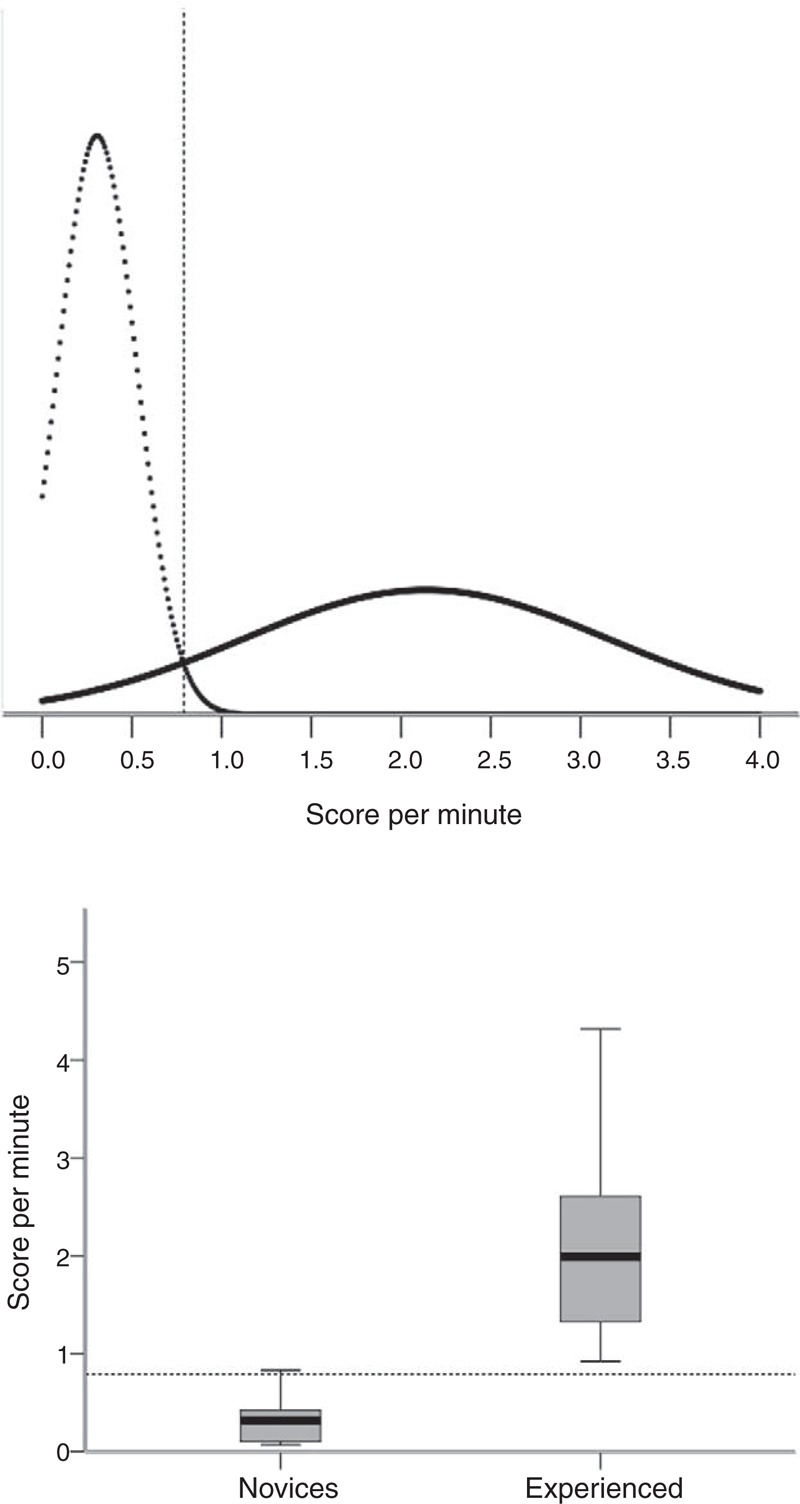
Phantom model test. (A) Establishing a pass/fail standard using the contrasting-groups method. The distributions of scores of novice endoscopists (dotted line, n = 15) and experienced endoscopists (solid line, n = 10) are shown. The pass score (0.79 points/min) was set at the intersection of the score distributions of the 2 groups. (B) Box plot showing the consequences of the established pass/fail criterion. All the experienced endoscopists passed the test as well as a single outlier in the novice group.

All tested metrics had discriminatory ability (Table 3). In the easy task, 1 of the novices, and in the more challenging task, 6 novices did not reach the cecum, both because of the cutoff time. All experienced endoscopists completed both the tasks. The mean aggregated score per minute in the novice group was 0.32 (SD 0.31) and for the experienced group was 2.48 (SD 1.09), *P* < 0.001 (Figure 2A)—ICC, average measures = 0.87, single measure = 0.77. The pass/fail standard was set to 0.79 points/minute. One novice managed to pass the test (Figure 2B).

**TABLE 3:**
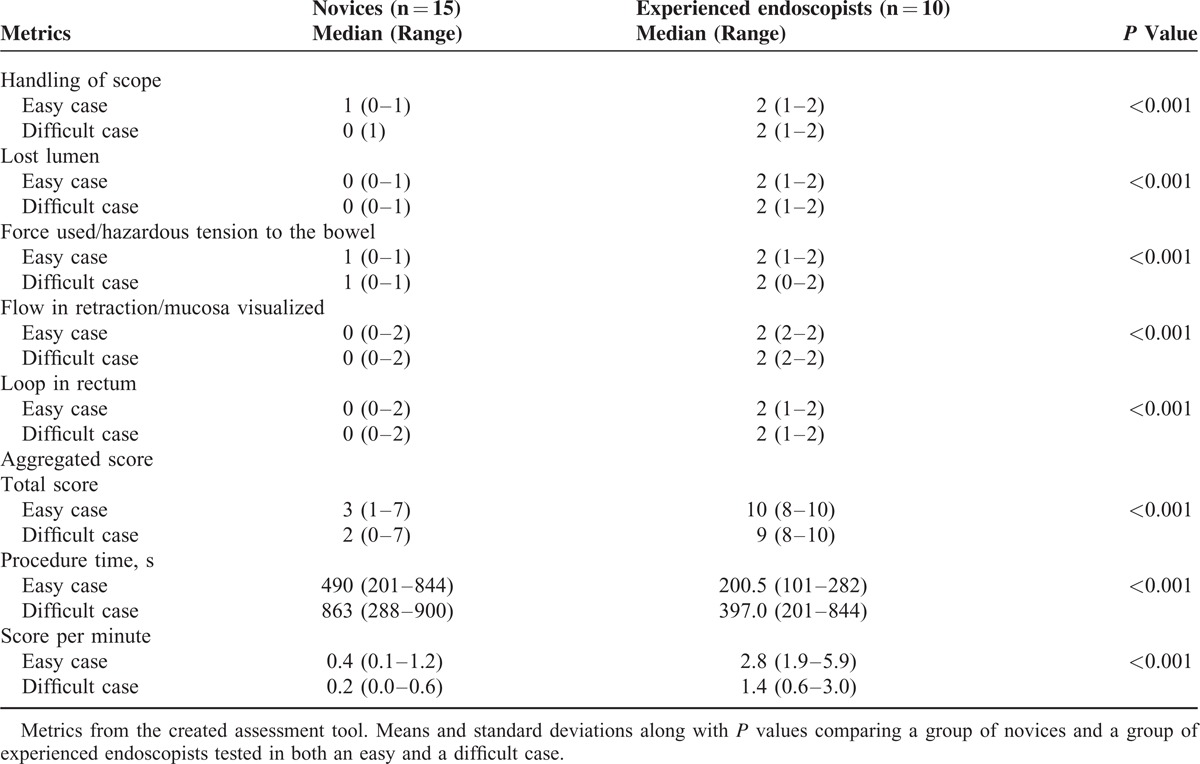
Metrics in the Phantom Model Test

## DISCUSSION

We explored 2 tests based on different simulator models in order to create a pass/fail criterion for each test. Our tests demonstrated construct validity with significant differences between novices and experienced endoscopists. In the virtual-reality simulator, we used the metrics supplied by the simulator and tested them for discriminatory ability to create an aggregated score. In the phantom model, we created an assessment tool and tested the metrics for discriminatory ability to create an aggregated score. Other tools have been made for bedside assessment of colonoscopy skills. However, advanced and validated tools such as DOPS^13^ and MCSAT^11^ are designed for clinical assessment in a patient setting by testing a branch of domains relevant to colonoscopy including communication, sedation, and safety that is relevant in the clinical training, whereas our aim was to create a simple assessment tool dedicated to assess the technical motor skills of colonoscopy in the simulator room.

### Virtual-Reality Simulator Test

In the virtual-reality simulator test, we found the following metrics to have discriminatory ability: total procedure time, intubation of terminal ileum, time to cecum, and percentage of mucosa visualized. In a review by Ansell et al^19^ looking at 13 studies validating colonoscopy simulators as assessment or training tools (12 virtual-reality studies and 1 animal model), it was reported that these exact metrics were the most valid training and assessment endpoints across all studies.

We found highly significant differences between the time to reach cecum in each group and the ability to perform intubation of terminal ileum. For time to cecum, there was significant difference between the 2 groups, but compared with similar studies, our novices were remarkable faster,^20^ which might be because of the fact that our novices had 30 minutes of formal supervised training before the test. The time difference was more significant in the easy case compared with the more difficult case, which could be explained by the fact that the experienced performer will do the easy case easily and fast, but when it comes to the more challenging case, it will take longer to perform; the novices, however, spent a long time on both the cases.

We found that testing in a single task did not provide a reliable test result—ICC single measure = 0.67. To ensure a reliability above 0.80,^21^ it was necessary to use 2 tasks—ICC average measures = 0.80. At least 2 procedures should be assessed when using the virtual-reality simulator for certification purposes.

### Phantom Model Test

Defining more direct competency endpoints has proven to be difficult in the absence of objective means to assess the core skills of colonoscopy.^16^ Handling of scope, safe scope advancement, force used, efficiency of withdrawal, and ability to loop in rectum all added to the aggregated score. There were significant differences between the 2 groups and all metrics showed discriminatory ability. Completion rates were consistent with the varying difficulty of the cases. In the easy case, 1 novice, and in the more difficult case, 6 novices failed to reach the cecum. All experienced participants completed both the cases. This difference was expected as cecal intubation rates correlates to experience.^11^

Regarding procedure time, both cases showed large group differences, as the experienced endoscopists were significantly faster than the novices. When a participant failed to complete a case, we used the maximum time allowed (15 minutes) to create the aggregated score. Differences might have been even larger if participants had been given an unlimited time to complete each case. Testing in 2 cases was necessary to ensure reliability—ICC average measures = 0.87, single measure = 0.77. At least 2 procedures should be assessed when using the phantom model for certification.

In an era of best evidence medical education, there is an increasing demand for evidence-based training programs and standards for evaluation. Our pass/fail standards showed discriminatory ability and construct validity.

Earlier simulator training studies have defined the amount of training based on hours spent in the simulator. The time spent differs from 3, 6, or 10, to even 20 hours of training^3,22–25^; however, individual trainees learn colonoscopy at different rates. Learning curves described by Marshall^6^ showed significant differences in time for reaching competence. In programs with a fixed number of hours or procedures in a simulator, there is the possibility that some students do not reach the adequate level of competence and others having already reached this level early in the program do not advance further. Schindler et al^26^ described the impact of using a standard-setting method for determining pass/fail scores in a surgery clerkship and found that standard-setting methods can be applied to a final clerkship grade even when multiple performance measures are used.

Simulators have become an integrated part of many training programs. In a review of 109 studies, Issenberg et al^27^ concluded that high-fidelity medical simulations are educationally effective and simulation-based education models complement medical education in patient care settings.

The fidelity of simulators is never perfect as the simulator environment differs in several ways from the clinical setting. Both tested models are regarded as high-fidelity simulators (close to real-life situations) and yet several limitations should be recognized. Hill et al^28^ tested 4 different simulator models for realism, including the 2 models in this study. The comparisons were based on a questionnaire tool. There was a significant difference between the realism of the virtual-reality models compared with the phantom models in favor of the phantom models.^28^ The virtual-reality simulators tend to lack the tactile experience and the elasticity of the colon and gut wall. The phantom models generate a more naturalistic experience. The difference between the 2 groups was bigger in the phantom model than in the virtual-reality simulator (equal to a smaller area under the overlapping curves in Figures 1 and 2, respectively), which could support a higher fidelity of the phantom model.

The phantom model does not generate any automatic feedback. Mahmood and Darzi^29^ published a study analyzing the effect of feedback in the simulator. Without feedback, they could not show any progress in training. Virtual-reality simulators provide feedback through performance metrics regarding the endoscopic view but they do not provide feedback regarding handling of the scope, use of knobs, or body posture.

Disadvantages of the virtual-reality simulators are that they are very expensive and require costly periodic software updates. The phantom models are cheaper, but require additional equipment (processor, monitor, and colonoscope) and regular cleaning procedures. Details on the 2 simulators including pros and cons are listed in Table 4

**TABLE 4:**
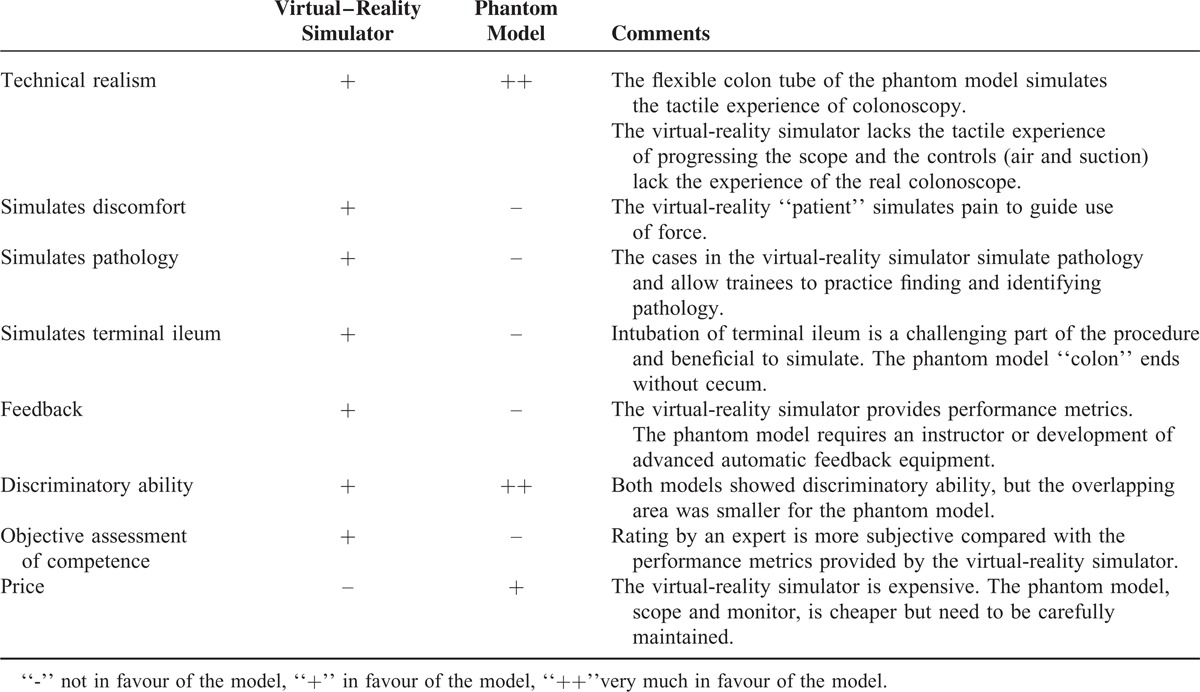
Comparative Overview of the Features of the 2 Tested Simulator Models

## LIMITATIONS AND FUTURE STUDIES

Our study has several limitations. Sample size was small (n = 25), but we explored 2 simulation-based tests with 2 tasks each (a total of 100 procedures), and demonstrated significant discriminatory ability between the 2 groups and good reliability of both the tests.

The risk of bias was the greatest limitation to the phantom model test, because of the fact that a single expert performed rating. A recent work by Konge et al^30^ comparing results based on direct observations and blinded video recordings showed a significant bias in nonblinded observations in favor of the consultants. To improve the level of evidence, blinded raters could be used; however, this method is costly with regard to time and equipment.

Another possibility is to look into nonbiased observations. Only few attempts at generating nonbias real-time assessment of phantom model simulators have been made. Plooy et al^31^ introduced an indirect measure of peak force using a force plate interposed between the table and the colon model and showed construct validity of their method. In a clinical setting, a recent study by Filip^32^ tested a software (colometer) for real-time assessment of colonoscopy, and further studies is needed to validate this approach. Use of a scope guide has shown better completion rates in trainees,^33^ and future studies are needed to see if a 3-D imager in combination with the scope guide could provide automatic nonbiased assessment of performance.

Being aware of the risk of bias, we chose our design because we wanted to create an operational tool, which could be easily implemented in any simulation center.

We recognize the limitations of simulator technologies. Simulator performance is never completely realistic and does not mirror all aspects of the clinical procedure. Our tests were developed with a focus on technical skills. However, other core skills of colonoscopy are not being measured by these tools such as cognitive knowledge of procedure and indications, pathology recognition, pain control, and safety of the patient. These important competencies must be assessed under direct observation. In our study, both models made it possible to distinguish between novices and experienced endoscopists but the distributions of scores within the groups were not identical comparing the virtual-reality model to the phantom model. The explanation could be that the 2 models test different procedural skills but could also represent an age difference in the confidence with computer animation.

## CONCLUSION

We established credible pass/fail standards for 2 colonoscopy competency tests based on a virtual-reality simulator and a phantom model, respectively. Both models are suitable for early simulator training in a colonoscopy training program with clearly defined outcomes and benchmarks for the learners.

## Acknowledgments

The authors would like to thank Dr Klaus Holtug, Dr Hans Jørgen Gyrtrup, Dr Bo Søndergaard, Dr Jørn Pacler, Dr Orhan Bulut, Dr Paul Krohn, Dr Charlotte Bulow, Dr Jakob Holm, and Dr Lene Brink for their kind participation in the experienced group.
